# Improvement of a synthetic lure for *Anopheles gambiae *using compounds produced by human skin microbiota

**DOI:** 10.1186/1475-2875-10-28

**Published:** 2011-02-08

**Authors:** Niels O Verhulst, Phoebe A Mbadi, Gabriella Bukovinszkiné Kiss, Wolfgang R Mukabana, Joop JA van Loon, Willem Takken, Renate C Smallegange

**Affiliations:** 1 Laboratory of Entomology, Wageningen University and Research Centre, P.O. Box 8031, 6700 EH, Wageningen, The Netherlands; 2 International Centre of Insect Physiology and Ecology, Mbita Point Research and Training Centre, P.O. Box 30, Mbita Point, Kenya; 3 School of Biological Sciences, University of Nairobi, P.O. Box 30197 - 00100 GPO, Nairobi, Kenya

## Abstract

**Background:**

*Anopheles gambiae sensu stricto *is considered to be highly anthropophilic and volatiles of human origin provide essential cues during its host-seeking behaviour. A synthetic blend of three human-derived volatiles, ammonia, lactic acid and tetradecanoic acid, attracts *A. gambiae*. In addition, volatiles produced by human skin bacteria are attractive to this mosquito species. The purpose of the current study was to test the effect of ten compounds present in the headspace of human bacteria on the host-seeking process of *A. gambiae*. The effect of each of the ten compounds on the attractiveness of a basic blend of ammonia, lactic and tetradecanoic acid to *A. gambiae *was examined.

**Methods:**

The host-seeking response of *A. gambiae *was evaluated in a laboratory set-up using a dual-port olfactometer and in a semi-field facility in Kenya using MM-X traps. Odorants were released from LDPE sachets and placed inside the olfactometer as well as in the MM-X traps. Carbon dioxide was added in the semi-field experiments, provided from pressurized cylinders or fermenting yeast.

**Results:**

The olfactometer and semi-field set-up allowed for high-throughput testing of the compounds in blends and in multiple concentrations. Compounds with an attractive or inhibitory effect were identified in both bioassays. 3-Methyl-1-butanol was the best attractant in both set-ups and increased the attractiveness of the basic blend up to three times. 2-Phenylethanol reduced the attractiveness of the basic blend in both bioassays by more than 50%.

**Conclusions:**

Identification of volatiles released by human skin bacteria led to the discovery of compounds that have an impact on the host-seeking behaviour of *A. gambiae*. 3-Methyl-1-butanol may be used to increase mosquito trap catches, whereas 2-phenylethanol has potential as a spatial repellent. These two compounds could be applied in push-pull strategies to reduce mosquito numbers in malaria endemic areas.

## Background

Host-seeking mosquitoes are mainly guided by chemical cues released by their blood hosts [1,2]. Some of these cues have already been identified for the malaria mosquito *Anopheles gambiae sensu stricto *(hereafter referred to as *A. gambiae*) and include ammonia, lactic acid and carboxylic acids [3-7], which are released from the human skin. These compounds are more attractive in mixtures than when applied alone [3,4]. Lactic acid, for example, is only slightly attractive on its own [5], but when combined with ammonia and carboxylic acids, this combination shows a synergistic effect [4].

Blends of ammonia, lactic acid and carboxylic acids have been shown to be attractive in the laboratory [4,6], and in semi-field and field set-ups when carbon dioxide (CO_2_) was added [3,8]. However, a blend of ammonia, lactic acid and carboxylic acids is still less effective than humans odours, when compared at close distance [3,9] and its attractive effect can probably be improved by the addition of other compounds [6].

Another chemical cue that plays an important role in mosquito host-seeking behaviour, including that of *A. gambiae*, is CO_2 _[10,11]. In the field, trap catches increase when CO_2 _is added to an odour blend [8,12-15].

Humans are differentially attractive to mosquitoes [16-21] and focusing on these differences can reveal new compounds that mediate the mosquito host-seeking process [20,21]. Analyses of human skin emanations, however, often result in hundreds of compounds [22,23], which makes identification of active compounds laborious, as each compound potentially may contribute to the overall attraction of the emanations. Recently it was shown that volatiles released by human foot bacteria grown *in vitro *attract *A. gambiae *[24]. Chemical analysis of the headspace collected from the cultures of these skin bacteria narrowed down the number of putative attractants to fourteen. A synthetic blend consisting of ten of these was attractive to *A. gambiae *[24], although not as attractive as the volatiles released by the skin bacteria themselves [[24], NO Verhulst, unpublished data]. In addition to this, when tested in semi-field experiments in Kenya, traps baited with CO_2 _and the blend of compounds did not catch more mosquitoes than the control traps baited with CO_2 _[NO Verhulst, unpublished data]. Possibly the concentrations of the chemicals tested were too low to attract the mosquitoes from a distance or some of the compounds in the blend acted as inhibitors, masking the attractive effect of the other components in the blend.

The purpose of the current study was to test the effect of each of the ten compounds present in the headspace of human foot bacteria on the host-seeking process of *A. gambiae*. Compounds may be more attractive in mixtures than when applied alone [4,25] and therefore the effect of each of the ten selected compounds on the attractiveness of a blend of ammonia, lactic acid and tetradecanoic acid [6] to *A. gambiae *was examined. Experiments were performed in an olfactometer and in a semi-field set-up to compare results obtained under laboratory and semi-field conditions.

## Methods

### Mosquitoes

The *Anopheles gambiae sensu stricto *colony used for the laboratory experiments originated from Suakoko, Liberia. Mosquitoes have been cultured in the Laboratory of Entomology of Wageningen University, The Netherlands, since 1988 and received blood meals from a human arm twice a week. Adult mosquitoes were maintained in 30-cm cubic gauze-covered cages in climate-controlled chambers (27 ± 1°C, 80 ± 5% RH, LD 12:12). They had access to a 6% (w/v) glucose solution on filter paper. Eggs were laid on wet filter paper and placed in tap water in plastic trays. Larvae were fed daily on Tetramin^® ^baby fish food (Melle, Germany). Pupae were collected daily and placed in 30-cm cubic cages for emergence.

The *Anopheles gambiae sensu stricto *colony at the Thomas Odhiambo campus of the International Centre of Insect Physiology and Ecology (ICIPE), Nyanza Province, western Kenya, was used for the semi-field assays. The colony originated from Mbita Point and has been cultured since 2001. Mosquitoes were fed three times a week on a human arm and larvae were reared in trays with filtered water from Lake Victoria. Adult mosquitoes had access to a 6% (w/v) glucose solution on filter paper. Eggs were laid on wet filter paper and placed in tap water in plastic trays. Larvae were fed daily on Tetramin^® ^baby fish food (Melle, Germany). Pupae were collected daily and placed in 30-cm cubic cages for emergence.

### Compounds

The ten compounds identified in previous experiments [24], were dispensed from sealed sachets (25 × 25 mm) of Low Density PolyEthylene sheet (LDPE; Audion Elektro, The Netherlands) [26]. Each sachet contained 100 μL of each of the ten diluted or undiluted compounds. In the laboratory experiments, compounds were tested in sachets of 0.20 mm sheet thickness and diluted in paraffin oil (Merck, Germany) (1:100; 1:1,000 or 1:10,000). In the semi-field experiments, compounds were tested undiluted in sachets of 0.20, 0.10 or 0.03 mm sheet thickness. The compounds 1-butanol, 2-methyl-1-butanol, 2-methylbutanal, 2-methylbutanoic acid, 3-hydroxy-2-butanone and 3-methylbutanoic acid were purchased from Sigma (Germany) and 2,3-butanedione, 3-methyl-1-butanol, 3-methylbutanol, 3-methylbutanal and 2-phenylethanol from Fluka (Germany). All compounds had purity levels between 95 and 99.8%.

For each of the ten compounds it was tested whether it increased or reduced mosquito catches of a basic blend of ammonia, L-(+)-lactic acid (henceforth termed lactic acid) and tetradecanoic acid [6] upon addition to the latter blend. Ammonia (100 μl of a 25% solution in water; analytical grade, Merck) and tetradecanoic acid (50 mg, >99%, Sigma) were released from separate LDPE sachets of 0.03 mm sheet thickness, while lactic acid (100 μl of a 88-92% aqueous solution, Riedel-de Haën) was released from a third LDPE sachet of 0.05 mm sheet thickness.

Carbon dioxide was added in the semi-field experiments as it has been shown to increase trap catches of *A. gambiae *under semi-field conditions [[14,27], NO Verhulst unpublished data]. Four compounds (2-methyl-1-butanol, 2-methylbutanal, 2-methylbutanoic acid and 3-methylbutanoic acid) were each tested with carbon dioxide (≥ 99.9%) provided from pressurized cylinders (Carbacid Investments Ltd., Kenya) (Table 1) through silicone tubing (Ø 7 mm; Rubber B.V., The Netherlands) connected to the Luer connection at the underside of the trap's top lid. The carbon dioxide was released at a rate of 500 ml/min regulated by a flow meter (Sho-Rate; Brooks Instruments, The Netherlands). The other six compounds (1-butanol, 2,3-butanedione, 3-hydroxy-2-butanone, 3-methyl-1-butanol, 3-methylbutanal and 2-phenylethanol) were each tested with yeast-produced CO_2 _(Table 1) as described by Smallegange *et al *[28]. Carbon dioxide was produced by mixing 17.5 g of dry yeast (Angel Instant Dry Yeast Co. Ltd., China), 250 g sugar (Sony Sugar, South Nyanza sugar Co. Ltd., Kenya) and 2.0 L tap water in a plastic bottle of 3 L, which results in a release rate of approximately 135 ml/min. Mixing took place 30 minutes before mosquitoes were released, at ambient temperature, until the dry yeast was dissolved.

**Table 1:** Response of *Anopheles gambiae *in an olfactometer to compounds identified in bacterial headspace samples.

Compound	Dilution	N	Treatment	Control	**χ**^**2**^**-test (P-value)**	Effect
1-butanol	1:100	167	34	39	0.56	
	1:1,000	160	23	38	0.05	
	1:10,000	163	32	17	0.03	+
2,3-butanedione	1:100	166	25	42	0.04	-
	1:1,000	166	25	39	0.08	
	1:10,000	169	44	28	0.06	
2-methyl-1-butanol	1:100	165	22	25	0.66	
	1:1,000	164	24	45	0.01	-
	1:10,000	168	38	22	0.04	+
2-methylbutanal	1:100	174	33	49	0.08	
	1:1,000	172	13	22	0.13	
	1:10,000	171	31	18	0.06	
2-methylbutanoic acid	1:100	167	49	25	0.01	+
	1:1,000	171	49	41	0.40	
	1:10,000	166	38	43	0.58	
3-hydroxy-2-butanone	1:100	168	21	29	0.26	
	1:1,000	170	29	36	0.39	
	1:10,000	170	36	17	0.01	+
3-methyl-1-butanol	1:100	163	29	31	0.80	
	1:1,000	158	20	28	0.25	
	1:10,000	157	41	25	0.048	+
3-methylbutanal	1:100	170	34	18	0.03	+
	1:1,000	168	24	21	0.65	
	1:10,000	172	16	16	1.00	
3-methylbutanoic acid	1:100	163	22	25	0.66	
	1:1,000	161	33	24	0.23	
	1:10,000	161	30	18	0.08	
2-phenylethanol	1:100	162	24	30	0.41	
	1:1,000	167	15	40	<0.001	-
	1:10,000	155	11	25	0.02	-

The ten test compounds were applied in three dilutions (1:100; 1:1,000; and 1:10,000) in LDPE sachets. The effect of the compounds on mosquito behaviour was examined by adding them individually to the attractive basic blend (treatment) and to test this combination against the basic blend (control). N = number of mosquitoes released. The effect (E) of the compound tested on the 'attractiveness' of the basic blend is indicated: + = significant increase of mosquito catches compared to the control, - = significant reduction of mosquito catches compared to the control.

The bottles were connected to the MM-X traps (American Biophysics Corp., USA) [29] using the original MM-X tubing (micron filter and orifice removed) and the Luer connection at the underside of the trap's top lid. The connections were sealed by Teflon tape to prevent leakage of carbon dioxide.

### Olfactometer experiments

A three layer dual-port olfactometer [30] was used to evaluate host-seeking responses of female mosquitoes to the ten compounds identified in a previous study [24]. Pressurized air was charcoal-filtered, humidified, and passed through two poly-methyl-methyl-acrylaat (PMMA or Perspex) mosquito trapping devices equipped with funnels [31], which were linked to both ports (diameter 5 cm, 25 cm apart) of the olfactometer. The air entered the flight chamber (1.50 × 0.50 × 0.50 m) at a speed of 0.21 ± 0.02 m/s. Temperature and humidity were recorded using data loggers (MSR145S, MSR Electronics GmBH, Switzerland). Temperature of the air entering the two trapping devices was 27.0 ± 1.2°C, and relative humidity was above 80%. The air temperature in the flight chamber was 25.8 ± 0.7°C and relative humidity was 77.3 ± 8.6%. The experimental room was maintained at a temperature of 25.8 ± 0.8°C and a relative humidity of 64.5 ± 5.1%.

Experiments were prepared and performed according to the methods described by Smallegange *et al *[4]. For each test 30 female mosquitoes of 5-8 d old, which had been provided a mating opportunity and not been offered a blood meal, were selected 14-18 h before the experiment and placed in a cylindrical release cage (d = 8 cm, l = 10 cm) with access to tap water from damp cotton wool. The experiments were performed during the last 4 h of the scotophase, when *A. gambiae *females are known to be highly responsive to host odours [32,33].

As another study in the olfactometer had found that undiluted compounds produced inhibitory effects, possibly because of the resulting high concentrations in the olfactometer flight compartment (G Bukovinszkiné Kiss and RC Smallegange, unpublished data), the compounds tested were diluted 1:100; 1:1,000 or 1:10,000 in paraffin oil. Each diluted solution of the volatile compounds was contained in a LDPE sachet and placed in the trapping device together with the three sachets, each containing one component of the basic blend (ammonia, lactic acid, tetradecanoic acid). The control trap was baited with the sachets making up the basic blend and one LDPE sachet containing 100 μl paraffin oil. All sachets were suspended by a hook as described before [24]. Clean air was tested against clean air, to test the symmetry of the system and to determine the mosquito response when no odour stimulus was present. The attractiveness of the basic blend was established by testing the three LDPE sachets containing ammonia, lactic acid and tetradecanoic acid against three sachets of the same size and sheet thickness, one empty and two filled with distilled water.

In each trial, test odours were released in the air stream before a group of mosquitoes was set free from a cage which was placed at the downwind end of the flight chamber, 1.50 m from the two ports. After 15 minutes mosquitoes that entered each of the two trapping devices were counted after anaesthesia with CO_2_. Each trial started with a fresh batch of mosquitoes, clean trapping devices, and new stimuli. Surgical gloves were worn by the researcher at all times to avoid contamination of equipment with human volatiles.

The sequence of test odours was randomized on the same day, between days and between the three layers of the olfactometers. Each treatment was repeated six times and test stimuli were alternated between right and left ports in different replicates to rule out any positional effects.

### Semi-field experiments

**Figure 1:**
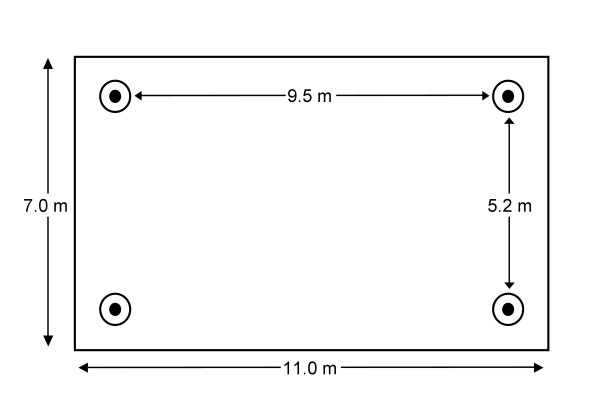
**Schematic drawing of semi-field set-up (top view)**. The rectangle represents the outline of the screened cage. Mosquitoes were released from the centre. Circles indicate the positions of the four MM-X traps.

Semi-field experiments were conducted at the Thomas Odhiambo campus of ICIPE, Kenya. Experiments were conducted as described before [27] in a greenhouse with a glass-panelled roof and gauze-covered side walls. Inside, sand covered the floor and a large mosquito-netting cage (11 × 7 × 2.5 m; mesh width 3 mm) was suspended from the ceiling to the floor. Four MM-X traps (American Biophysics Corp., USA) [29] were placed in the corners of the greenhouse (Figure 1), with the odour outlet positioned 15 cm above ground level [14,15]. MM-X traps were used because they have a high discriminatory power [14].

For each test 200 female mosquitoes, 3-6 d old that had been held together with male mosquitoes to allow mating and that had not received a blood meal, were selected 8 h before the experiments. Mosquitoes were placed in a 1 L (d = 11-13 cm, h = 15 cm) cup, covered by mosquito netting, and were offered water-moistened cotton wool only. Every test night, the mosquitoes were released from the centre of the greenhouse at 8.00 pm. At 6.30 am the following morning traps were collected and placed in a freezer, after which mosquitoes were counted. Every afternoon the mosquitoes remaining in the greenhouse were captured and the sand in the greenhouse moistened to prevent dust formation and to lower the temperature. Surgical gloves were worn by the researcher to avoid contamination of equipment with human volatiles.

Each of the four traps in the greenhouse was provided with CO_2 _(either from a cylinder or yeast-produced) and the basic blend released from three LDPE sachets containing either ammonia, lactic acid or tetradecanoic acid (see above). Three traps were provided with LDPE sachets containing the compound to be tested, each in a sachet of different thickness to test various release rates. Sachets were used with a sheet thickness of either 0.03 mm, 0.10 mm or 0.20 mm and release rates were measured by weighing (laboratory: AC100, Mettler, Germany; semi-field, A200S Sartorius, Germany; accuracy both 0.1 mg) the sachets before and after the experiments [26]. LDPE sachets were suspended by hooks in the black tube of the MM-X trap [24]. Treatments and traps were randomized over the four positions to complete 3 series of a 4 × 4 Latin square in 12 nights.

A data logger (TinyTag Ultra, model TGU-1500, INTAB Benelux, The Netherlands) recorded temperature and humidity during the experiments in the middle of the screenhouse, every ten minutes. During the nights of the semi-field experiments (March - September 2009), the average temperature was 23.6 ± 2.6°C and the average relative humidity 71.5 ± 18.4%.

### Statistics

To test whether the release rates of each compound varied exponentially with LDPE sheet thickness as described by Torr *et al *[26], the release rates of each compound and the LDPE thickness were fitted to an exponential regression line (Genstat, release 12.1).

For each two-choice test in the olfactometer a χ^2^-test was used to analyze whether the total (i.e. sum of all replicates) number of mosquitoes that was trapped in the treatment trapping device and the total number that was trapped in the control trapping device differed from a 1:1 distribution (P < 0.05).

A Generalized Linear Model (GLM, Binomial, logit link function, dispersion estimated) was used to investigate the relative attractiveness of each combination of odours tested in the traps in the semi-field experiments, expressed as the number of mosquitoes caught in one of the traps divided by the total number of mosquitoes trapped in all four traps during each experimental night [16,34]. Two-sided t-probabilities were calculated to test pairwise differences between proportions. Effects were considered to be significant at P < 0.05. For each series of 12 nights in which a compound was tested, the effect of CO_2 _source, trap location, temperature and humidity on mosquito catches was tested and fitted as parameters in the GLM model when significant. Another GLM (Binomial, logit link function, dispersion estimated) was used to test the effect of CO_2 _source (cylinder or yeast-produced) on catches of traps baited with the basic blend alone.

## Results

### Olfactometer experiments

The results of the olfactometer experiments in which no odour stimuli were placed in either of the trapping devices showed that the olfactometer was symmetrical in all three layers (χ^2^-test, d.f. = 1, P > 0.05). Trapping devices baited with the basic blend caught significantly more mosquitoes than trapping devices baited with LDPE sachets with water alone (χ^2^-test, d.f. = 1, P < 0.001). These results are in accordance with previous experiments [6].

Of the ten compounds tested, five compounds (1-butanol, 2-methylbutanoic acid, 3-hydroxy-2-butanone, 3-methyl-1-butanol and 3-methylbutanal) increased the number of mosquitoes caught in the trapping devices baited with the basic blend compared to the trapping devices baited with the basic blend alone (Table 1; χ^2^-test, d.f. = 1, P < 0.05). This depended, however, on the concentration tested (Table 1). Three compounds (2-methyl-1-butanol, 2,3-butanedione and 2-phenylethanol) caused fewer mosquitoes to enter the trapping devices compared to the trapping devices baited with the basic blend alone, dependent on the concentration tested (Table 1; χ^2^-test, d.f. = 1, P < 0.05). 2-Phenylethanol was the only compound that reduced the number of mosquitoes caught in the trapping devices with the basic blend at the two lowest concentrations tested (Table 1; χ^2^-test, d.f. = 1, 1:1,000 P < 0.001 and 1:10,000 P = 0.02). A concentration of 1:1,000 of 2-methyl-1-butanol reduced the number of mosquitoes caught in the trapping devices baited with the basic blend (χ^2^-test, d.f. = 1, P < 0.05), whereas a concentration of 1:10,000 of this compound led to an increased number of mosquitoes caught (Table 1).

### Semi-field experiments

An exponential correlation was found between LDPE thickness and release rates of six of the ten compounds tested (Table 2). The CO_2 _source used (pure CO_2 _from a pressurized cylinder or CO_2 _produced by fermenting yeast) had no significant effect on trap catches (GLM, d.f. = 1, P = 0.96). The spatial position of the trap in the greenhouse had a significant effect on the trap catches of all compounds tested (Table 3; GLM, d.f. = 3, P < 0.05), except in the case of 2-methyl-1-butanol (GLM, d.f. = 3, P = 0.54), and was therefore included in the GLM model. Treatment had a significant effect on trap catches with 2,3-butanedione, 3-hydroxy-2-butanone, 3-methyl-1-butanol, 3-methylbutanoic acid or 2-phenylethanol (Table 3; GLM, d.f. = 3, P < 0.05).

**Table 2:** Correlation between LDPE thickness and release rate of bacterial volatiles as determined by weight loss of the LDPE sachets.

			Exponential regression parameters A + B*(R^X^)		
	**R**^**2**^	P-value	A	B	R
1-butanol	12.8	0.06	0.00036	0.0200	5.22E-18
2,3-butanedione	64.3	<0.001	0.00387	0.0975	5.28E-13
2-methyl-1-butanol	_^1^	0.46	-0.00042	0.0052	1.49E-12
2-methylbutanal	31.7	<0.01	0.00709	0.0595	6.96E-13
2-methylbutanoic acid	2.6	0.26	0.01020	9.585E-19	6.275E+79
3-hydroxy-2-butanone	58.7	<0.001	0.000242	0.0122	1.27E-19
3-methyl-1-butanol	61.7	<0.001	0.0003722	8.561	2.307-125
3-methylbutanal	33.8	<0.001	0.00745	0.0495	2.88E-11
3-methylbutanoic acid	_^1^	0.85	0.01007	3.244E-19	5.125E+79
2-phenylethanol	35.3	0.001	0.001333	3.12E-19	6.80E+80

The release rate of each compound (Y, g/night) was fitted by an exponential regression model (A + B*(R^X^); X = LDPE thickness, mm) [26]. R^2 ^= coefficient of determination.

^1^Residual variance exceeds variance of response variate.

**Table 3:** Mean trap catches of *Anopheles gambiae *in a semi-field set-up to compounds identified in bacterial headspace samples.

			BB	0.03		0.10		0.20	
Compound	L	T	Mean ± SE	Mean ± SE	E	Mean ± SE	E	Mean ± SE	E
1-butanol	<0.001	0.10	38.4 ± 6.7	47.0 ± 9.5		37.4 ± 6.7		28.6 ± 4.9	
2,3-butane-dione	<0.001	0.02	29.0 ± 4.4	29.5 ± 6.3		55.2 ± 14.4	+	30.0 ± 7.2	
2-methyl-1-butanol	0.54	0.20	24.9 ± 4.3	23.2 ± 2.8		22.6 ± 3.5		33.4 ± 6.3	
2-methyl-butanal	0.04	0.07	29.6 ± 4.7	18.4 ± 3.3 -	-	30.1 ± 5.2		23.3 ± 3.9	
2-methyl-butanoic acid	0.001	0.93	19.6 ± 3.1	19.0 ± 4.6		18.0 ± 3.9		20.1 ± 4.3	
3-hydroxy-2-butanone	<0.001	<0.001	44.0 ± 9.3	52.8 ± 6.4		33.3 ± 5.8	-	27.8 ± 4.3	-
3-methyl-1-butanol	<0.001	<0.001	19.1 ± 3.3	29.1 ± 6.2		61.3 ± 9.6	+	29.8 ± 6.4	+
3-methyl-butanal	0.007	0.670	25.8 ± 7.2	22.3 ± 3.6		24.4 ± 7.1		18.9 ± 2.3	
3-methyl-butanoic acid	<0.001	<0.001	32.4 ± 4.1	11.9 ± 0.8	-	58.0 ± 5.8	+	13.0 ± 1.9	-

All traps were provided with CO_2 _and the basic blend (BB). To three traps a LDPE sachet of different thickness (0.03 mm, 0.10 mm or 0.20 mm) was added containing one of the 10 test compounds. The effect of trap location (L) and treatment (T) on the mean number of mosquitoes caught per night (± standard error) is given (GLM, y = location*x_1 _+ treatment*x_2_, P-values given). The effect of the compound tested on the 'attractiveness' of the BB is indicated: + = significant increase of mosquito catches compared to the BB, - = significant reduction compared to the BB (GLM, P < 0.05).

Traps to which either 2,3-butanedione, 3-methyl-1-butanol or 3-methylbutanoic acid was added caught significantly more mosquitoes than traps baited with the basic blend and CO_2 _alone, depending on the sachet sheet thickness used to release the test compound from (Table 3; GLM, d.f. = 3, P < 0.05). Traps baited with either 2-methylbutanal, 3-hydroxy-2-butanone, 3-methylbutanoic acid or 2-phenylethanol and the basic blend and CO_2 _caught significantly fewer mosquitoes than traps with the basic blend and CO_2_, depending on the sachet sheet thickness used to apply the test compound (Table 3; GLM, d.f. = 3, P > 0.05). 2-Phenylethanol was the only compound that reduced trap catches independent of the LDPE thickness tested, compared to the number of mosquitoes caught by traps to which no 2-phenylethanol was added (Table 3; GLM, d.f. = 3, P < 0.05).

## Discussion

The identification of volatiles produced by human skin bacteria narrows down the number of putative mosquito attractants emitted by humans [24]. The three-layer olfactometer and semi-field system allowed for high-throughput testing of these volatiles. Of the 10 compounds identified in the headspace of the human foot bacteria many caused a behavioural response of *A. gambiae *in both olfactometer and semi-field experiments, with an attractive or inhibitory effect, dependent on the compound and concentration tested.

Eight out of the 10 compounds tested in the olfactometer had a significant effect on the number of mosquitoes caught when compared with the numbers attracted to the basic blend only. Six of these significantly increased the number of mosquitoes caught with the basic blend when tested at a specific concentration. In the semi-field system, six out of the ten compounds tested had a significant effect on the trap catches when combined with CO_2 _and the basic blend and tested against CO_2 _and the basic blend alone. At certain concentrations, three of these compounds increased the 'attractiveness' of the basic blend combined with CO_2_.

The differences between the olfactometer and semi-field results indicate that probably the concentration at which each compound is tested has an important effect on the response of *A. gambiae*. This has also been observed in previous experiments with *A. gambiae *in laboratory and (semi-)field experiments [2-6,35]. As has been mentioned previously [6], it is difficult to estimate and compare the concentrations of the volatiles in the odour blends tested as encountered by the mosquitoes in the two different bioassays. The different results obtained with 2,3-butanedione, 3-hydroxy-2-butanone and 3-methylbutanal in the two bioassays show this clearly. This underlines the importance of control over the concentration tested and testing more than three concentrations in future experiments is likely to provide a better understanding of the effect of each compound [4]. Entrainment of volatiles that are released in the olfactometer and semi-field set-ups and determining the concentration of the volatiles by gas chromatography-mass spectrometry (GC-MS) will allow calculation of the average volatile concentrations encountered by the mosquitoes. The average concentrations will depend on: distance from the odour source (H Beijleveld, unpublished data), prevailing wind direction and speed in the semi-field set-up, temperature and humidity [26]. It is likely that the average odorant concentrations as used in the present olfactometer study were lower than those used in the semi-field facility [26]. Rather than average concentrations, however, instantaneous flux values resulting from encountering of volatile packages, are relevant for understanding orientation behaviour [36]. Notwithstanding the current lack of information on behaviourally active odour concentrations in both assay systems, previous work on the impact of aliphatic carboxylic acids on *A. gambiae *behaviour has shown that the pathway of odour discovery as used in these studies is valid, as several compounds that were attractive in the laboratory also proved attractive in the (semi-)field, even though volatile fluxes in the two bioassays were probably different since different release methods were used [6,3].

Of four compounds (1-butanol, 2-methyl-1-butanol, 2-methylbutanoic acid and 3-methylbutanoic acid) the release rates from the LDPE sachets in the semi-field experiments was very low or the variation too high to find a significant correlation with LDPE sheet thickness (Table 2), even though LDPE material was used in an attempt to standardise the release rate of each of the candidate compounds[31]. The use of LDPE tubes is expected to reduce variation in the release rate, depending on the compound tested [26], while the use of nylon strips or open glass vials might increase the release of compounds [37].

2-Methylbutanal reduced the attractiveness of the basic blend in the semi-field system at the highest concentration tested. In the olfactometer, the highest concentration also reduced the number of mosquitoes caught, although the effect was marginally significant (Table 1, P = 0.08). 2-Phenylethanol clearly inhibited mosquito attraction exerted by the basic blend as its addition reduced the number of mosquitoes caught by more than 50% in both bioassays. Future experiments with 2-phenylethanol may elucidate its potential as a spatial repellent for personal or household protection.

3-Methyl-1-butanol increased the number of mosquitoes caught with the basic blend in both experimental set-ups when tested at the lowest concentrations. In the semi-field, traps baited with 3-methyl-1-butanol applied in a LDPE sachet of 0.10 mm sheet thickness together with the basic blend and CO_2 _caught three times more mosquitoes than traps baited with the basic blend and CO_2 _alone. A recent field study has shown that synthetic odour blends can compete with natural host odour when placed in separate huts [3] and the results obtained with the combination of 3-methyl-1-butanol, the basic blend and CO_2 _are encouraging as a novel attractant for future use in malaria vector monitoring or reduction.

Carbon dioxide was used in the semi-field experiments, but not in the olfactometer experiments, which could explain some of the differences between the results obtained in the two set-ups. In the olfactometer, CO_2 _was not added because previous results had not shown an effect of this addition [38,39]. Carbon dioxide was added in the semi-field experiments as it has been shown to increase trap catches of *A. gambiae *under these conditions [[14,27], NO Verhulst unpublished data]. Carbon dioxide was added from two different sources: either from a pressurized cylinder containing pure CO_2 _or from a bottle containing fermenting yeast. The two methods resulted in different release rates of CO_2_, which might have affected the outcome of the experiments. In addition to CO_2 _yeast also produces other organic compounds and in a previous study, traps baited with yeast-produced CO_2 _caught more mosquitoes than traps baited with CO_2 _supplied from cylinders [28]. In the current study, however, no difference was found in trap catches between the cylinder and yeast-produced CO_2 _when added to the basic blend, although CO_2 _concentrations used were different from the previous study. For field application the use of yeast-produced CO_2 _is preferable as it has several advantages above CO_2 _from cylinders: it is cheaper, easier to handle and easier to obtain [28].

## Conclusions

To date only a limited number of compounds that have an impact on the host-seeking behaviour of *A. gambiae *have been identified [2]. The identification of volatiles released by human skin bacteria resulted in a selection of 10 compounds of which eight had an effect on host-seeking behaviour in the laboratory and six in the semi-field. Two compounds showed a similar result in both bioassays and in multiple concentrations. 2-Phenylethanol reduced, whereas 3-methyl-1-butanol increased the attractiveness of the basic blend in both set-ups.

Carbon dioxide produced by yeast-sugar solutions applied from bottles, together with ammonia, lactic acid, tetradecanoic acid and 3-methyl-1-butanol applied in LDPE sachets can be an easy to use, cost-effective combination for monitoring and possible reduction of *A. gambiae *populations. In so-called push-pull systems [3,15,20,40-42], the synthetic odour blend including 3-methyl-1-butanol may be used to 'pull' mosquitoes into traps. 2-Phenylethanol is a candidate compound to be added as a spatial repellent to 'push' mosquitoes away from human dwellings.

## Competing interests

The authors declare that they have no competing interests.

## Authors' contributions

The initial experimental designs were developed by NOV, RCS, JJAL and WRM. GBK and NOV conducted the behavioural experiments in the laboratory, PAM the semi-field experiments. NOV drafted the manuscript. RCS, WT, JJAL and WRM contributed to drafting the final manuscript. All authors read and approved the final manuscript.
